# Distinct patterns of adult transport-related physical activity (TRPA) behaviour exist independent of the TRPA behaviours of childhood: the childhood determinants of adult health study

**DOI:** 10.1186/s12966-023-01462-w

**Published:** 2023-05-26

**Authors:** Jack T. Evans, Marie-Jeanne Buscot, Seana Gall, Terence Dwyer, Alison Venn, Verity Cleland

**Affiliations:** 1 Menzies Institute for Medical Research, University of Tasmania, Tasmania, Australia; 2 School of Clinical Sciences, Monash University, Melbourne, Australia; 3 Nuffield Department of Women’s & Reproductive Health, University of Oxford, Oxford, UK; 4 Murdoch Children’s Research Institute, Melbourne, Australia; 5 School of Exercise and Nutrition Sciences, Deakin University, Burwood, Australia; 6 17 Liverpool St, Hobart, TAS 7000 Australia

**Keywords:** Commuting, Public health, Transportation, Physical activity

## Abstract

**Background:**

Transport-related physical activity (TRPA) is recognised as a potential means of increasing total physical activity participation that may yield substantial health benefits. Public health campaigns focusing on promoting TRPA from a young age aim to develop life-long healthy habits. However, few studies have examined how TRPA changes across the lifecourse and whether childhood TRPA levels influence those observed later in life.

**Methods:**

Using the Australian Childhood Determinants of Adult Health study (baseline, 1985), latent class growth mixture modelling with adjustment for time-varying covariates was performed using four timepoints (ranging from 7 to 49 years) to assess behavioural patterns and retention of TRPA across the lifecourse. As child and adult adjusted TRPA measures could not be harmonised, trajectories of adult TRPA (n = 702) were instead identified, and log-binomial regression analysis was performed to determine whether childhood levels of TRPA (high/medium/low) influenced these trajectories.

**Results:**

Two stable groups of adult TRPA trajectories were identified: persistently low (n = 520; 74.2%), and increasingly high TRPA (n = 181; 25.8%). There was no significant relationship between childhood TRPA levels and patterns in adulthood (relative risk of high childhood TRPA yielding high adult TRPA trajectory membership = 1.06; 95% confidence interval = 0.95–1.09).

**Conclusion:**

This study found childhood TRPA levels were not associated with TRPA patterns in adulthood. These findings suggest that while TRPA in childhood may have health, social, and environmental benefits, it does not appear to impact adult TRPA directly. Therefore, further intervention is required beyond childhood to promote the implementation of healthy TRPA behaviours into adulthood.

**Supplementary Information:**

The online version contains supplementary material available at 10.1186/s12966-023-01462-w.

## Introduction

Physical activity (PA) is a key behaviour for the prevention and management of chronic disease [1, 2]. Despite being readily modifiable [3], a large proportion of the population (approximately 81% of adolescents and 23% of adults globally) do not meet daily PA recommendations [4]. Total physical activity is accumulated across four domains: leisure, transport, domestic, and occupational. Of these domains, transport-related physical activity (TRPA) and leisure-time physical activity are considered predominantly discretionary in nature and are therefore often the target of strategies to increase total PA. Examination of the aetiology and epidemiology of physical activity in separate domains of physical activity could provide important insights into the patterns and predictors of specific domains. Examination of individual domains, particularly those that are discretionary and hence modifiable, may facilitate better tailored and targeted interventions that may therefore be more effective.

TRPA has been identified as a potential mechanism for increasing total PA participation and yielding considerable health benefits. Prior systematic review and meta-analyses have found greater TRPA was associated with decreased cardiovascular risk [5] and all-cause mortality [6] when adjusting for total PA. TRPA encompasses active travel behaviours such as walking and cycling as a means of transport and can be undertaken independently or together with additional public or private transportation.

Compared to the study of total and leisure-time PA little examination of TRPA has occurred, particularly longitudinally [7]. The tracking of total and leisure-time PA across the lifecourse has been examined in detail, with trajectory and life-period analysis showing PA to track from childhood to adulthood [8–10]. Comparatively, lifecourse patterns of TRPA remain poorly understood. Previously, the Childhood Determinants of Adult Health (CDAH) study, examined total PA and its individual domains from childhood and young-adulthood and found no significant association between child and adult TRPA [11]. While these analyses provide insight into longitudinal relationships, the prior analysis only used data from two timepoints and did not consider the role of time-varying covariates across the lifecourse. As such, the potential for different contributions of predictors towards associations at different timepoints and trajectory divergence throughout the lifecourse were potentially overlooked.

Literature examining the factors that shape TRPA behaviour are primarily cross-sectional in nature. A systematic review of the cross-sectional correlates and longitudinal determinants of adult TRPA found 36 factors have been commonly examined, seven of which demonstrate consistent associations (socio-economic status, self-efficacy, social normalisation, distance of travel, destinations, public transportation, and the presence of streetlighting) [12].

Despite this lack of longitudinal research, public health campaigns focus on the promotion of TRPA from a young age aim to develop healthy habits for retention across the lifecourse. Examples include initiatives such as the United States “Let’s Move”[13] and “Safe Routes to School” [14] programs aimed at helping children develop healthy habits that may be retained into the future. In Australia, the “Walk to School” program encourages children to undergo an active commute to school to build healthy travel behaviours and achieve national PA guidelines [15, 16]. While it is established that some healthy habits that are learnt and reinforced throughout childhood inform behaviours in later life [17], few studies have examined how TRPA changes across the lifecourse. As such, the impact of childhood TRPA behaviours on adult TRPA outcomes remains relatively unknown.

It is important to understand whether TRPA behaviours formed in childhood are retained, or whether behaviours fluctuate over time. While the retention of childhood behaviours may suggest childhood intervention is warranted, a lack of lifecourse relationship would demonstrate that the immediate needs and circumstances of the individual may exert predominant influence over their TRPA behaviours. Absence of association between childhood and adulthood TRPA would highlight the need for period specific intervention in order to promote TRPA amongst adults whose TRPA and total PA levels are insufficient. Therefore, this analysis aimed to identify TRPA patterns across the lifecourse into mid-adulthood and whether childhood TRPA predicts lifecourse TRPA patterns.

## Methods

### Study design and participants

Participants for this analysis were drawn from the ongoing longitudinal Childhood Determinants of Adult Health (CDAH) study. A prospective cohort of Australian adults spanning over 30 years, the CDAH study is a continuation of the 1985 Australian Schools Health and Fitness Survey (ASHFS). Procedures describing the selection of participants at baseline have been outlined in detail elsewhere [18]. ASHFS was a representative sample of 8498 Australian children aged 7–15 years who underwent physical, behavioural, and anthropometric assessments. A flowchart detailing CDAH Study participation is displayed in the supplementary material (Additional file 1, Figure S1). At baseline, ethical approval was granted by Directors of Education of each state, consent was provided by schools and parents, and children gave assent. Ethical clearance at follow-up was obtained from the Southern Tasmanian Medical Research Ethics Committee (CDAH-1: H0008152, CDAH-2: H0010454, CDAH-3: H0013826) with participants providing written informed consent. A STROBE checklist of items for cohort studies is presented within the supplementary material (Additional file 1, Table S1).

### Measurements

#### Childhood transport-related physical activity

At baseline, participants aged 9–15 years completed questionnaires in small groups supervised by trained study data collectors. Participants were asked separate questions regarding the duration (“how many hours and minutes were spent each session?”), the frequency (“how many times last week?”), of travel to and from school via bicycle and walking. For each mode of transport, minutes per week performing these activities was calculated [19].

#### Adult transport-related physical activity

TRPA was assessed at CDAH-1 (aged 26–36 years; n = 2700), CDAH-2 (aged 31–41 years; n = 1649), and CDAH-3 (aged 36–49 years; n = 1794) via completion of the International Physical Activity Questionnaire (IPAQ) [20]. Participants reported TRPA duration and frequency across the past week, from which minutes per week of TRPA was then calculated.

#### Covariates

Detailed methods regarding the measurement of covariates are provided within Supplementary Material. At baseline, covariates of age, sex, self-reported health, smoking status, body mass index, area-level socio-economic status derived from residential postcode, and teacher-rated scholastic ability were recorded. During adult CDAH follow-ups the aforementioned covariates were assessed with the addition of highest education level, employment status, occupation type, number of children, and marital status.

### Statistical analyses

Latent Class Growth Mixture Modelling (LCGMM), a form of trajectory analysis, was performed with the initial intention of assessing the behavioural patterns and retention of TRPA from childhood across the life-course to mid-adulthood. This technique allows the examination of patterns of changes in TRPA across the lifecourse. However, as will be demonstrated in this paper, the use of trajectory analysis has its own unique challenges. Here, we detail the process that led to the modelling of adult TRPA trajectories and the analysis of their association with childhood TRPA.

#### The latent class analysis approach

Traditional longitudinal methods (individual-based approaches, including hierarchical modelling) assume that every participant’s trajectory follows a similar pattern representative of the average trajectory of the population and use covariates to explain variability about this average. In this framework, individual deviations from this mean population longitudinal trend can be represented in terms of individual-level random effects, indicating how individual parameters of change differ from the population-average parameters. This simplification yields a single homogeneous population-average trajectory with a single estimate of variance. Contrastingly, LCGMM is a group-based trajectory analysis technique that considers that a given population consists of several heterogeneous subpopulations with distinct trajectory patterns [21]. Data is used to infer the number of distinct trajectories (class enumeration), their unique shapes, and the number of individuals probabilistically assigned to each identified trajectory (i.e., trajectory membership). The use of data driven clustering allows this technique to overcome issues of misclassification and information loss associated with the subjective clustering of participants into trajectory groups defined *a priori* [21]. One of the practical difficulties of this approach is that the LCGMM model is unable to adjust for time-varying covariates such as body mass index, education, and number of children. Adjustment for potential confounding at each timepoint is crucial, as these covariates influence the repeated measures of TRPA at each follow-up. To determine whether TRPA observed across the lifecourse is a result of behaviours developed in childhood and retained with age rather than a reflection of the circumstances participants experience at each follow-up, four phases of analysis were undertaken.

##### Phase 1- covariates and TRPA residuals extraction

**Fig. 1:**
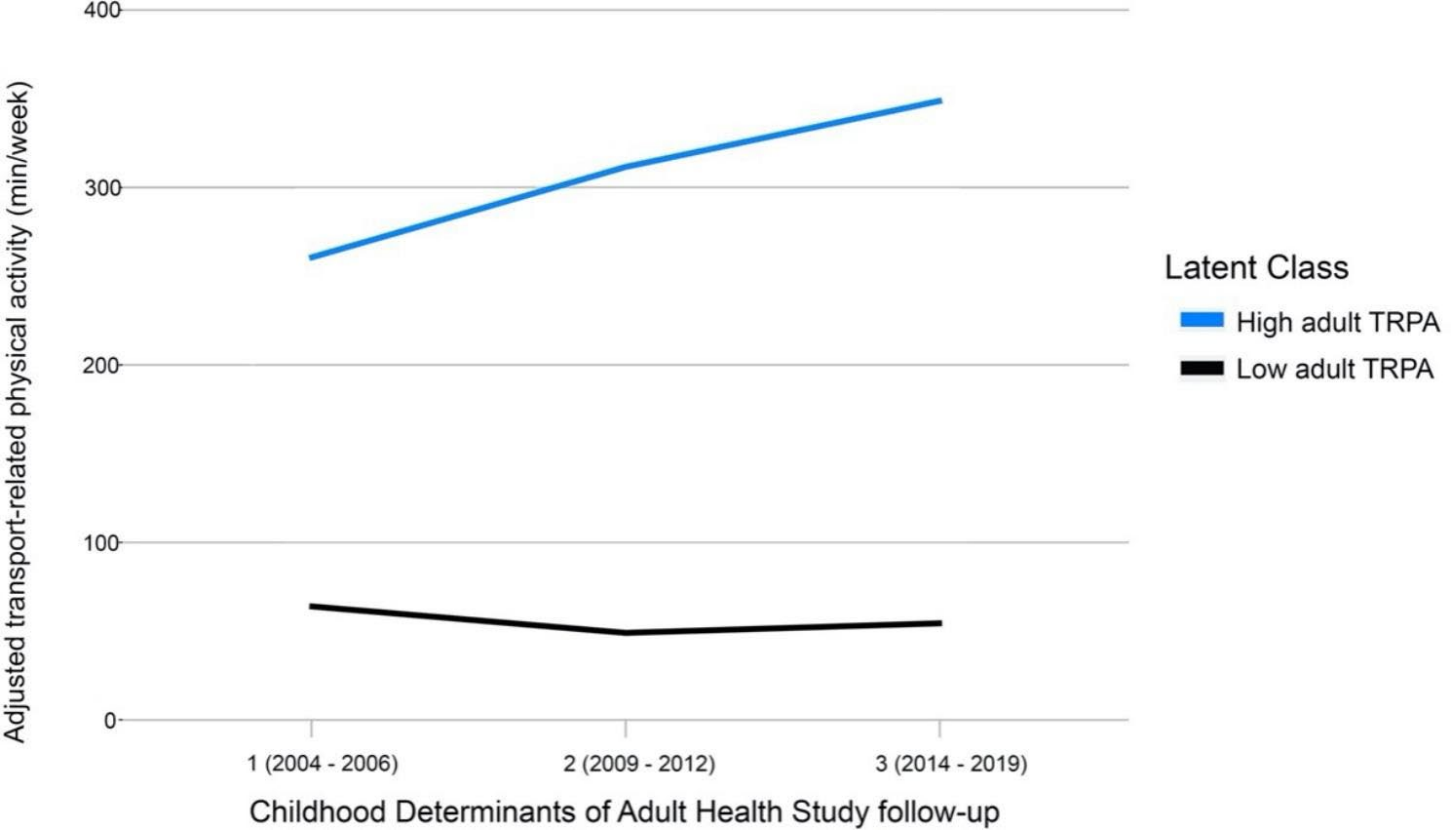
Distinct latent patterns of adjusted transport-related physical activity identified from early to mid-adulthood in the Childhood Determinants of Adult Health study Solid lines show class-specific mean adjusted transport-related physical activity levels predicted using a two-class growth mixture model

Previous literature indicates that a number of individual-level correlates influence TRPA levels in adulthood [12]. Exploratory modelling of changes in observed TRPA across the lifecourse showed there to be erratic movement of TRPA among individuals with no discernible systematic pattern of change. Therefore, to examine TRPA independent of exogenous factors, TRPA was adjusted for covariates at each time point. Separate linear models were fit at each timepoint, regressing total PA levels and all covariates against the outcome of TRPA. Residuals were extracted for each TRPA observation, representing the effect unexplained by the relationship between the exposures (covariates) and TRPA at each of those timepoints, theoretically providing a measure of the TRPA undertaken independent of covariates at each timepoint. Residuals at each timepoint were extracted only from individuals with observations present for covariates assessed (Fig. 1).

##### Phase 2 – exploration, issues, and modification of approach

Following adjustment of TRPA for covariates at each timepoint, further data exploration suggested changes over time in TRPA among individuals. Preliminary LCGMM was then performed. As TRPA was collected using different questionnaires in childhood (ASHFS) and adulthood (CDAH) these measures of adjusted TRPA were not directly comparable in the context of the LCGMM analysis. Childhood measurements of TRPA only took into consideration activity undertaken in the commute to-and-from school, while adult TRPA was representative of all TRPA undertaken for any utilitarian transportation purposes. As such, even when using standardised measures, the differences in variance and mean values between child and adult TRPA meant that modelling child and adult TRPA together yielded implausible trajectory results in our preliminary analyses.

As it was not possible to harmonise child and adult TRPA measures, this study instead identified TRPA trajectories through the observed adult life-period where TRPA was measured in the same way and then aimed to determine whether a relationship existed between childhood TRPA levels and these trajectories.

##### Phase 3 – LCGMM analysis of TRPA residuals

Trajectory analysis of adult TRPA was performed across adult CDAH timepoints (CDAH-1, CDAH-2, and CDAH-3) to determine whether patterns of TRPA behaviour existed among the population. A series of LCGMM considering linear, and quadratic polynomial specifications of TRPA residuals as a function of timepoint were fit. The model with the best fit was selected based on goodness of fit, Akaike information criteria (AIC), and Bayesian information criteria (BIC) indices (as described by Proust-Lima [22]). Additionally, the proportion of participants assigned to each class with a posterior probability > 0.8, and values of mean posterior class membership probabilities were assessed to select the best fitting model. Model building and class enumeration was undertaken following recommendations of Proust-Lima [22].

Seven-hundred-and-two participants with adjusted TRPA outcomes were included within this analysis. Selection of model specifications and number of classes is detailed within the supplementary materials (Table S2).

##### Phase 4 – Assessment of childhood TRPA as a predictor of adult TRPA trajectory

**Fig. 2:**
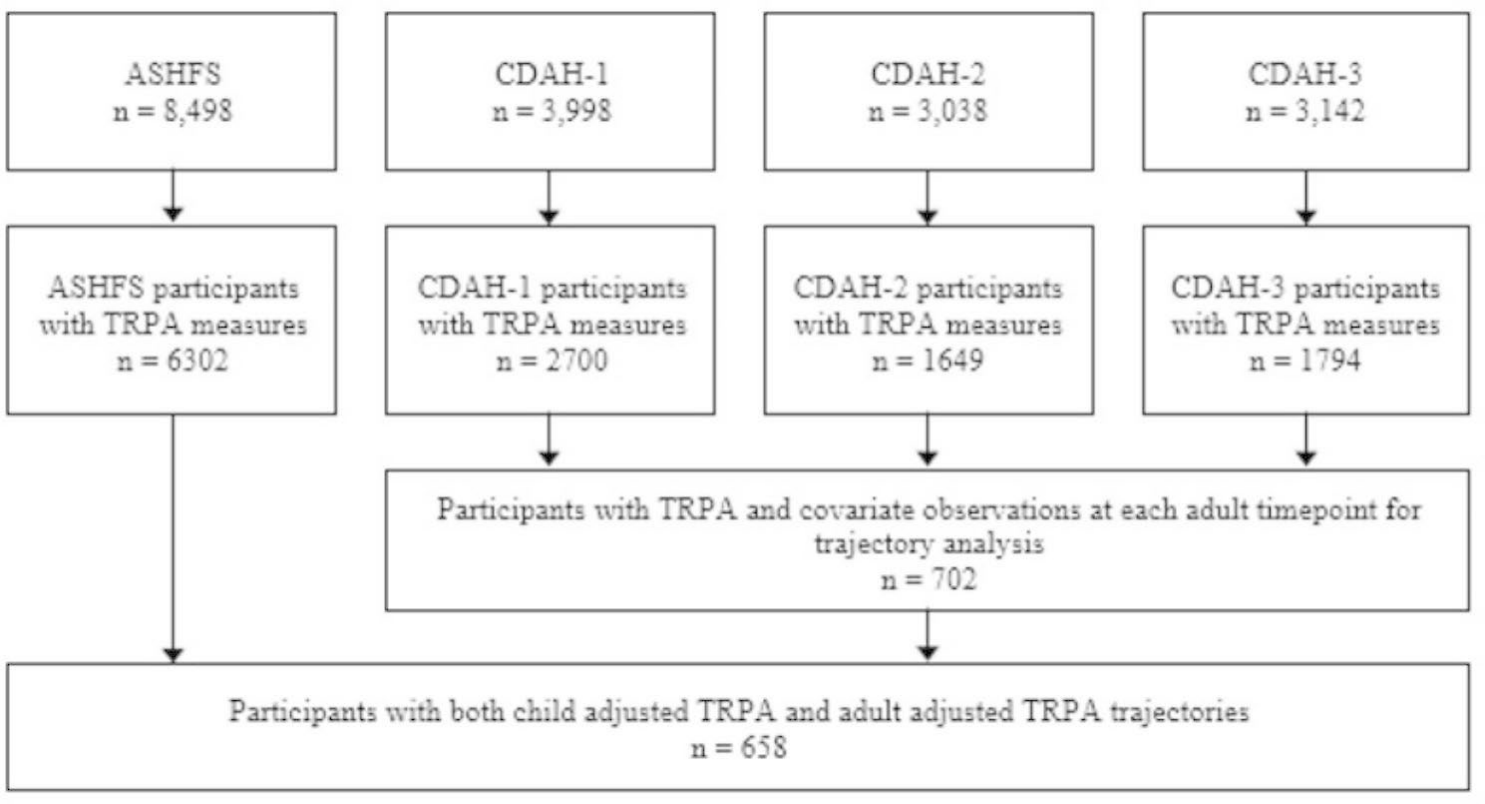
Flowchart of participant inclusion for analysis

Following the classification of adult TRPA patterns, we then examined whether adult TRPA behaviours were associated with an individual’s childhood TRPA level. As an adjusted measure of childhood TRPA (as described above) was used and there are no domain-specific physical activity guidelines to guide cut-point selection, classification of a tertiary measure could not be based upon common thresholds of activity. As such, adjusted TRPA was instead stratified at the 33rd and 66th percentiles to form a tertiary measure. Those who fell in the top third of the sample were considered to have high levels of childhood adjusted TRPA at that timepoint. To determine the relationship between childhood adjusted TRPA level and patterns of adult TRPA, log-binomial regression of adjusted childhood TRPA (i.e., low/medium/high) against the outcome of adult TRPA latent class membership (e.g., class 1, class 2, etc.) was performed amongst 658 participants with observations of both adjusted childhood TRPA and adult TRPA class membership. Additional analyses of continuous adjusted childhood TRPA and adult TRPA latent class membership is presented in the supplementary material. A flowchart of participant inclusion for analyses is presented in Fig. 2. Linear and log-binomial regression, residual extraction, and LCGMM analyses were performed using R (version 3.5.3; R Foundation for Statistical Computing, Vienna, Austria) and RStudio (version 1.2.1511; RStudio, Inc, Boston, MA, USA) using the package lcmm 1.9.3.

## Results

### Participant characteristics

Mean participant age at ASHFS was 12 years (Table 1). At follow-up, mean age for CDAH-1 participants was 32.1 years, CDAH-2 was 37.0 years, and CDAH-3 mean age was 44.5 years. Body mass index was observed to increase over time on average. Across all adult timepoints, more than one third of participants had high education levels.

**Table 1:** Characteristics of study population

	ASHFS N = 658	CDAH-1 N = 702	CDAH-2 N = 702	CDAH-3 N = 702
Age, mean (SD)	11.9 (2.0)	32.0 (2.1)	36.9 (2.1)	44.5 (2.2)
Sex (male), % (n)	47.6 (313)	47.3 (332)	47.3 (332)	47.3 (332)
Body mass index (kg/m^2^), mean (SD)	18.5 (2.6)	26.0 (4.8)	26.6 (4.7)	28.0 (5.5)
Self-reported health, % (n)	N = 658	N = 673	N = 377	N = 633
Excellent	37.2 (245)	17.1 (115)	15.1 (57)	18.8 (119)
Very good	42.6 (280)	43.7 (294)	43.5 (164)	43.1 (273)
Good	19.3 (127)	32.5 (219)	34.0 (128)	31.0 (196)
Fair & Poor	0.9 (6)	6.7 (45)	7.4 (28)	7.1 (45)
Teacher-reported scholastic ability, % (n)	N = 658			
Excellent	13.1 (86)			
Very good	36.5 (240)			
Good	38.3 (252)			
Fair	10.5 (69)			
Poor	1.7 (11)			
Area-level socio-economic status, % (n)	N = 658			
Low	7.0 (46)			
Medium low	35.2 (231)			
Medium high	29.3 (193)			
High	28.5 (187)			
Highest education level, % (n)		N = 676	N = 599	N = 520
High		40.2 (272)	42.6 (255)	46.5 (242)
Medium		31.2 (211)	32.4 (194)	49.8 (259)
Low		28.6 (193)	25.0 (150)	3.7 (19)
Occupation, % (n)		N = 678	N = 376	N = 517
Managers/professionals		55.3 (347)	53.5 (201)	61.1 (316)
White collar		15.0 (94)	19.7 (74)	14.1 (73)
Blue collar		17.8 (112)	15.7 (59)	14.9 (77)
Not in labour force		11.9 (75)	11.1 (42)	9.9 (51)
Marital status, % (n)		N = 702	N = 377	N = 526
Single		35.9 (252)	15.7 (59)	10.1 (53)
Married/partnered		61.8 (434)	79.3 (299)	81.1 (426)
Divorced/separated		2.3 (16)	5.0 (19)	8.8 (46)
Widowed		0 (0)	0 (0)	0.2 (1)
Children, mean (SD)		1.6 (1.1)	1.6 (1.3)	2.0 (1.2)
Smoking duration, % (n)	N = 658			
“I don’t smoke”	90.1 (593)			
“Just started”	2.1 (14)			
1–6 months	1.8 (12)			
7–12 months	1.4 (9)			
1–2 years	2.4 (16)			
>2 years	2.2 (14)			
Smoking frequency, % (n)		N = 675	N = 376	N = 495
Never		53.9 (364)	53.2 (200)	57.2 (283)
Ex-smoker		24.4 (165)	31.1 (117)	32.7 (162)
Less than weekly		2.8 (19)	1.1 (4)	0.6 (3)
Weekly		2.8 (19)	1.1 (4)	1.4 (7)
Daily		16.0 (108)	13.5 (51)	8.1 (40)
Transport-related physical activity (mins/week), median (IQR)	24.0 (0.0–80.0)	50.0 (0.0-140.0)	60.0 (0.0-180.0)	60.0 (0.0-164.0)

ASHFS = Australian Schools Health and Fitness Survey,

CDAH = Childhood Determinants of Adult Health Study, SD = Standard Deviation

### Lifecourse transport-related physical activity trajectories

The LCGMM identified two distinct trajectories of adjusted TRPA (residuals of linear regression of covariates on TRPA at each timepoint) throughout adulthood (Fig. 1). Using adjusted TRPA, the LCGMM yielded classes representative of the proportion of variation in TRPA not explained by time-varying covariates. The two distinct trajectories were qualitatively labelled as ‘persistently low’ adult TRPA (n = 520; 74.2%) and ‘high/ increasing’ adult TRPA (n = 181; 25.8%). Characteristics of participants within each trajectory are presented within Table 2.

**Table 2:** Characteristics of participants within each transport-related physical activity trajectory

	Transport-related physical activity trajectory	
	Low (N = 520)	High (N = 181)
**ASHFS Baseline**		
Sex (male), % (n)	46.3 (241)	50.3 (91)
Age, mean (SD)	11.9 (2.0)	12.0 (2.1)
Body mass index (kg/m^2^), mean (SD)	18.5 (2.6)	18.6 (2.6)
Self-reported health, % (n)		
Excellent	35.8 (186)	39.8 (72)
Very good	44.6 (232)	37.6 (68)
Good	19.0 (99)	20.4 (37)
Fair & Poor	0.6 (3)	2.2 (4)
Education - Teacher-reported scholastic ability, % (n)		
Excellent	13.4 (66)	12.4 (21)
Very good	37.2 (183)	34.3 (58)
Good	37.8 (186)	39.6 (67)
Fair	10.0 (49)	11.8 (20)
Poor	1.6 (8)	1.8 (3)
Smoking duration, % (n)		
“I don’t smoke”	90.3 (467)	88.9 (160)
“Just started”	2.5 (13)	1.1 (2)
1–6 months	1.7 (9)	2.2 (4)
7–12 months	1.7 (9)	1.1 (2)
1–2 years	2.2 (11)	2.8 (5)
>2 years	1.6 (8)	3.9 (7)
Area-level socio-economic status, % (n)	N = 506	N = 175
Low	5.7 (29)	12.0 (21)
Medium low	37.7 (191)	33.1 (58)
Medium high	28.5 (144)	26.9 (47)
High	28.1 (142)	28.0 (49)
Transport-related physical activity (mins/week), median (IQR)	25.0 (0.0–80)	30.0 (0.0–92.0)
**CDAH-3**		
Age, mean (SD)	44.6 (2.1)	44.5 (2.2)
Body mass index (kg/m^2^), mean (SD)	27.9 (5.5)	28.2 (5.5)
Self-reported health, % (n)		
Excellent	19.9 (93)	15.8 (26)
Very good	40.8 (189)	50.9 (84)
Good	32.5 (152)	26.7 (44)
Fair & Poor	7.1 (33)	6.6 (11)
Highest education level, % (n)		
High	46.7 (178)	46.4 (64)
Medium	49.9 (190)	50.0 (69)
Low	3.4 (13)	3.6 (5)
Occupation, % (n)		
Managers/professionals	61.6 (234)	60.3 (82)
White collar	14.7 (56)	12.5 (17)
Blue collar	14.2 (54)	16.9 (23)
Not in labour force	9.5 (36)	10.3 (14)
Marital status, % (n)		
Single	9.6 (37)	11.4 (16)
Married/partnered	81.0 (313)	80.7 (113)
Divorced/separated/widowed	9.4 (35)	7.9 (11)
Children, mean (SD)		
Smoking frequency, % (n)	2.0 (1.2)	1.8 (1.3)
Never	59.3 (217)	51.2 (66)
Ex-smoker	30.9 (113)	38.0 (49)
Less than weekly	0.5 (2)	0.8 (1)
Weekly	9.2 (34)	10.0 (13)
Transport-related physical activity (mins/week), median (IQR)	30.0 (0.0–90.0)	315.8 (163.6–420.0)

ASHFS = Australian Schools Health and Fitness Survey,

CDAH = Childhood Determinants of Adult Health Study, IQR = Inter-quartile range, SD = Standard Deviation

### Childhood and adulthood adjusted transport-related physical activity

**Fig. 3:**
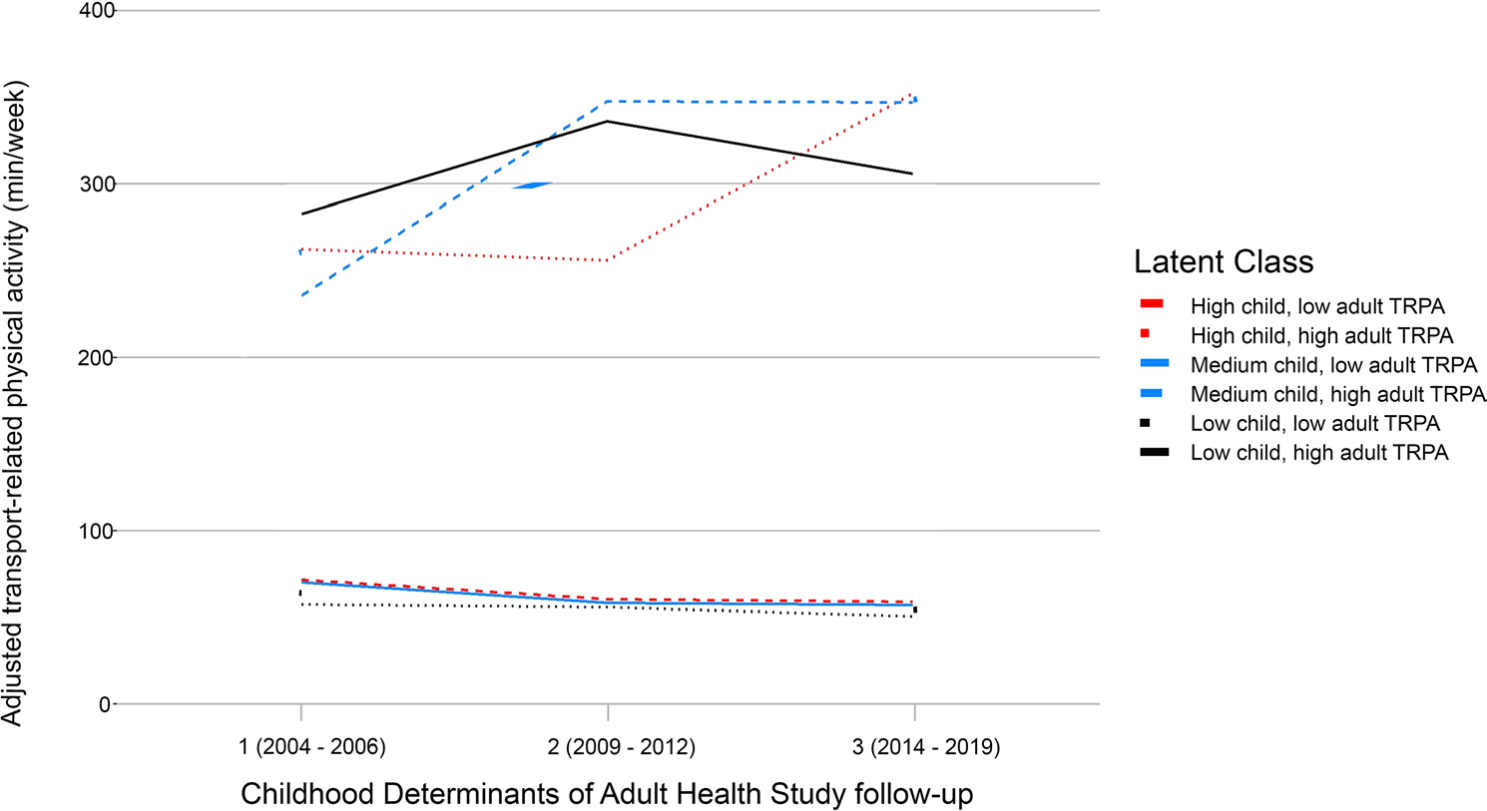
Adult transport-related physical activity latent classes stratified by tertiles of childhood transport-related physical activity level in the Childhood Determinants of Adult Health study

Log-binomial regression of adjusted childhood TRPA level against adult TRPA class found no significant relationship between childhood TRPA and adulthood TRPA (Low adjusted childhood TRPA [reference], Medium [relative risk (RR) = 1.04; 95% confidence interval (CI) = 0.94–1.17], and High [RR = 1.06 ;95%CI = 0.95–1.09]). Similarly, stratification of the latent classes by tertiary childhood TRPA illustrated that TRPA tracked across adulthood via the two trajectories of high/increasing and persistently low with minimal difference in both magnitude and shape, irrespective of childhood values (Fig. 3). Findings of non-significance were further reflected in the supplementary analysis of continuous adjusted childhood TRPA against adult TRPA class (Supplementary Material).

## Discussion

In a sample of Australian children followed into mid-adulthood over three decades, we found two distinct classes of adult TRPA: persistently low and high/increasing. Our findings of two distinct groups show that independent of key individual-level covariates (e.g., age, sex, education, occupation), TRPA remains an underlying behaviour that tracks across adulthood. No significant association was observed between child and adult TRPA behaviours. This lack of association was reflected in the visualisation of adult TRPA patterns stratified by childhood TRPA level. Stratified sub-classes showed minimal deviation from the adult TRPA patterns (persistently low and high/increasing) derived from LCGMM. These findings suggest that adult TRPA is not reflective of childhood behaviours and is instead reflective of more immediate individual circumstances.

While studies have shown the prevalence of TRPA among individuals to decline with age [9], to our knowledge only one study has previously examined the association between child and adult TRPA levels, observing childhood TRPA levels to not be associated with adult TRPA [11]. The findings of the current study support and expand upon these observations. While prior examinations of TRPA have been undertaken using traditional means of longitudinal assessment, this analysis is the first to assess patterns of TRPA across adulthood while adjusting for time-varying covariates. Using LCGMM, our results strengthen the literature finding that the relationship between childhood TRPA level and patterns of TRPA behaviour across the adult life-period is minimal.

This study’s findings of no significant association between child and adult TRPA behaviours are perhaps unsurprising. With extensive evidence showing relationships between the built environment and TRPA [12], it is possible that the effect of the factors such as perceived safety, travel distance and number of destinations may overcome any potential effect of learnt behaviours and negate any longitudinal association. Future study is suggested to examine if relationships between child and adulthood TRPA would alter when specifically adjusting for built environmental covariates and examining not only TRPA behaviours, but those relating to public transport use also.

While a lack of longitudinal relationship does not diminish the need to encourage childhood TRPA [23], it does highlight a need for interventions and policies targeting TRPA in both the child and adult population. With the majority of the sample population (73%) found to undertake low levels of adult TRPA, the importance of continued TRPA intervention is further emphasised. Prior studies have found TRPA to provide a considerable contribution to overall PA and the achievement of international PA recommendations [24, 25]. Our observations of distinct patterns of adult TRPA highlight potential to increase overall adult PA levels through initiatives aimed at supporting those with low TRPA participation to increase their TRPA.

TRPA differs to many of the outcomes that trajectory analysis is typically applied to (e.g., body mass index [21]) as there are no clear ‘underlying age-related’ progressions in this behaviour. Ultimately, as TRPA may be influenced by instantaneous predictors rather than a deterministic ‘growth’ process, adjustment for predictors is crucial to determine whether a trajectory exists. Without these considerations, the application of LCGMM and other trajectory analyses to outcomes without clear growth processes may yield misleading results. This study demonstrates a means in which future studies may overcome issues associated with assessing atypical measures for the presence of underlying trajectories.

A number of potential limitations are noted within this study. The childhood measure of TRPA presented in this study was comprised only of PA undertaken whilst walking or cycling to school. As such, childhood TRPA may not be completely representative of total TRPA as children during this period may have also used public transport (PT) (e.g., trains, trams, and buses) in their commute. Literature describing the relationship between childhood and adult PT use is sparce, therefore it is unknown whether adult PT behaviours are reflective of those in childhood. As a large component of adult TRPA is likely formed from PT use, the comparison of child and adult TRPA behaviours may be limited.

Differential loss-to-follow-up was observed among participants across study follow-ups. Comparison of baseline characteristics found that those excluded from the LCGMM analysis (N = 7480) were an average of one year younger and of a lower scholastic ability than those included (Supplementary Material, Table S3) As those lost to follow-up often exhibit poorer health behaviours, it is possible that the findings of this study may underestimate the proportion of people that may fall within the ‘persistently low’ class. Missing data among participants reduced the sample size. This study does not have observations of the social and built environment in which the participants reside. As prior studies have found there to be a number of factors associated with adult TRPA at the individual, social, and environmental level [12], the unavailability of social and environmental covariates prevents this study from providing a truly independent assessment of TRPA behaviours. Further examination is warranted to understand whether patterns of behaviour may differ among populations within environments supportive of TRPA (e.g., higher perceived safety, greater destination density, higher public transport frequency), compared to those considered unsupportive (e.g., greater travel distance and fewer destinations, fewer streetlights). Further, the use of self-reported measurement of TRPA limits this study due the potential for recall error, additionally the IPAQ only asks about TRPA with minimum bouts exceeding 10 min, therefore overlooking TRPA of shorter duration [26].

This study was strengthened through its use of LCGMM. This approach allows *a posteriori* determination of distinct trajectories without the risk of misclassification and information loss that often occurs when classes are defined *a priori* [21]. Additionally, the inclusion of regression residuals as LCGMM outcomes allowed models to be adjusted for the effect of time-varying covariates. This resulted in the first assessment of adult TRPA patterns that were resultant of underlying behaviours and habits rather than the TRPA resultant of known TRPA correlates and determinants.

## Conclusion

This study found that childhood TRPA levels were not associated with TRPA patterns experienced in adulthood when adjusted for individual-level covariates. Furthermore, TRPA behaviours tracked across adulthood with two stable TRPA trajectory groups, participants with persistently low, and those with high/increasing TRPA, independently of individual-level time-varying covariates. Identification of these two trajectories highlights an opportunity to better understand the characteristics of those whose TRPA is persistently low in adulthood, providing insights for intervention development. These findings suggests that while TRPA interventions targeting children may be beneficial to the development of TRPA behaviours during that age-period, further intervention is required to promote the implementation of healthy TRPA behaviours in adulthood.

## Electronic supplementary material

Below is the link to the electronic supplementary material.

Supplementary Material 1

## Data Availability

The datasets used and/or analysed during the current study available from the corresponding author on reasonable request.

## Abbreviations

ASHFS: Australian schools health and fitness survey
CDAH: Childhood determinants of adult health study
CI: Confidence Interval
IPAQ: International physical activity questionnaire
IQR: Interquartile range
LCGMM: Latent class growth mixture modelling
PA: Physical activity
PT: Public transportation
RR: Relative risk
SD: Standard deviation
TRPA: Transport-related physical activity
