# Supplementary material for: Distinct patterns of adult transport-related physical activity (TRPA) behaviour exist independent of the TRPA behaviours of childhood: the childhood determinants of adult health study
Source: Int J Behav Nutr Phys Act. 2023 May 26;20:63. doi: 10.1186/s12966-023-01462-w (PMC10214540; doi:10.1186/s12966-023-01462-w)
Supplement: Supplementary file 1 — Supplementary Material 1 [file 12966_2023_1462_MOESM1_ESM.docx]

**Additional File 1. Supplementary Material**

**
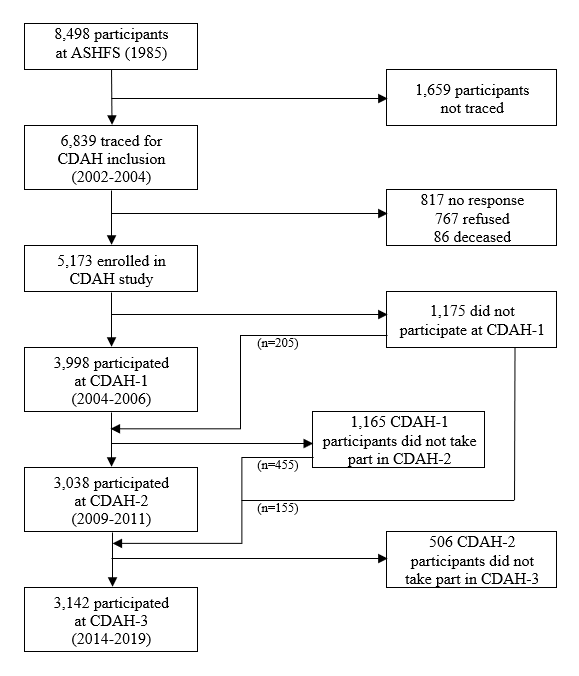
**

**Figure S1.** Flowchart of Childhood Determinants of Adult Health study participation

**Table S1. STROBE Statement—Checklist of items that should be included in reports of *cohort studies***

|  | Item No | Recommendation | Page |
| --- | --- | --- | --- |
| **Title and abstract** | 1 | (*a*) Indicate the study’s design with a commonly used term in the title or the abstract | 1 |
|  |  | (*b*) Provide in the abstract an informative and balanced summary of what was done and what was found | 2 |
| Introduction | | |  |
| Background/rationale | 2 | Explain the scientific background and rationale for the investigation being reported | 3-4 |
| Objectives | 3 | State specific objectives, including any prespecified hypotheses | 4 |
| Methods | | |  |
| Study design | 4 | Present key elements of study design early in the paper | 5 |
| Setting | 5 | Describe the setting, locations, and relevant dates, including periods of recruitment, exposure, follow-up, and data collection | 5 |
| Participants | 6 | (*a*) Give the eligibility criteria, and the sources and methods of selection of participants. Describe methods of follow-up | 5-6 |
|  |  | (*b*) For matched studies, give matching criteria and number of exposed and unexposed | n/a |
| Variables | 7 | Clearly define all outcomes, exposures, predictors, potential confounders, and effect modifiers. Give diagnostic criteria, if applicable | 5-7 |
| Data sources/ measurement | 8* | For each variable of interest, give sources of data and details of methods of assessment (measurement). Describe comparability of assessment methods if there is more than one group | 5-7 |
| Bias | 9 | Describe any efforts to address potential sources of bias | 10 |
| Study size | 10 | Explain how the study size was arrived at | 5-6, 10-12 |
| Quantitative variables | 11 | Explain how quantitative variables were handled in the analyses. If applicable, describe which groupings were chosen and why | 6-12 |
| Statistical methods | 12 | (*a*) Describe all statistical methods, including those used to control for confounding | 8-12 |
|  |  | (*b*) Describe any methods used to examine subgroups and interactions | n/a |
|  |  | (*c*) Explain how missing data were addressed | 9-10 |
|  |  | (*d*) If applicable, explain how loss to follow-up was addressed | 9-10 |
|  |  | (*e*) Describe any sensitivity analyses | 12, S6 |
| Results | | |  |
| Participants | 13* | (a) Report numbers of individuals at each stage of study—eg numbers potentially eligible, examined for eligibility, confirmed eligible, included in the study, completing follow-up, and analysed | 5-6, 11-12  Fig.1, Fig.2 |
|  |  | (b) Give reasons for non-participation at each stage | Fig.1 |
|  |  | (c) Consider use of a flow diagram | Fig.1, Fig.2 |
| Descriptive data | 14* | (a) Give characteristics of study participants (eg demographic, clinical, social) and information on exposures and potential confounders | 12, Table 1 |
|  |  | (b) Indicate number of participants with missing data for each variable of interest | Table 1 |
|  |  | (c) Summarise follow-up time (eg, average and total amount) | 5 |
| Outcome data | 15* | Report numbers of outcome events or summary measures over time | 10, Table 1 |
| Main results | 16 | (*a*) Give unadjusted estimates and, if applicable, confounder-adjusted estimates and their precision (eg, 95% confidence interval). Make clear which confounders were adjusted for and why they were included | Fig.1,  Fig.2,  14-15 |
|  |  | (*b*) Report category boundaries when continuous variables were categorized | n/a |
|  |  | (*c*) If relevant, consider translating estimates of relative risk into absolute risk for a meaningful time period | n/a |
| Other analyses | 17 | Report other analyses done—eg analyses of subgroups and interactions, and sensitivity analyses | n/a |
| Discussion | | |  |
| Key results | 18 | Summarise key results with reference to study objectives | 16 |
| Limitations | 19 | Discuss limitations of the study, taking into account sources of potential bias or imprecision. Discuss both direction and magnitude of any potential bias | 17 |
| Interpretation | 20 | Give a cautious overall interpretation of results considering objectives, limitations, multiplicity of analyses, results from similar studies, and other relevant evidence | 16-18 |
| Generalisability | 21 | Discuss the generalisability (external validity) of the study results | 16-17 |
| Other information | | |  |
| Funding | 22 | Give the source of funding and the role of the funders for the present study and, if applicable, for the original study on which the present article is based | 19 |

*Give information separately for exposed and unexposed groups.

**Measurement of covariates**

At baseline, date of birth and sex were reported via questionnaire. Age (years) was then calculated from date of birth at each follow-up. Childhood area-level socio-economic status was categorised into quartiles at ASHFS (low, medium-low, medium-high, and high), estimated from participant residential postcodes using the Australian Bureau of Statistics Socio-economic Index for Areas (SEIFA) (1). Self-reported health at baseline and follow-ups was reported as excellent, very good, good, fair, and poor. At baseline, smoking status was categorised by duration: “I don’t smoke,” “just started”, “1-6 months,” “7-12 months,” “1-2 years,” and “>2 years.” At CDAH follow-ups smoking was reported by frequency: “never smoked,” “ex-smoker,” “less than weekly,” “weekly,” and “daily.” Body mass index (BMI) was (kg/m^2^) calculated as body mass (kg) divided by height (m) squared. Participants’ height was assessed using a KaWe height tape (KaWe Kirchner & Wilhelm, Asperg, Germany) at the 1985 childhood baseline and a Leicester height measure (Invicta, Leicester, UK) in adulthood. Body mass was measured via beam or medical spring scales at baseline and Heine scales (Heine, Dover, NH) at adult CDAH follow-ups. In childhood, teachers reported scholastic ability (1=excellent – 5=poor). Highest level of education attained was categorically assessed at CDAH follow-ups via questionnaire as high- (higher degree, post graduate diploma, bachelor’s degree), medium- (undergraduate diploma, associate diploma, skilled and basic vocational training), and low- (primary and high school education only) education. Employment status was self-reported as employed or unemployed while occupation was categorised as manager/professionals, white collar (clerical, sales, service), blue collar (trades, labour), and not in labour force (students, unemployed, retired, home duties). Number of children was self-reported at each CDAH follow-up. Marital status was assessed at CDAH follow-ups via questionnaire as single, married/living as married relationships, divorced/separated, and widowed.

**Latent-class model fit**

The latent class model estimation was performed using the *lcmm* package in R. Prior to final model selection, various LGCMM models were fit and compared. When fitting the model across the three adult timepoints, two possible polynomial specifications (linear and quadratic) were considered for the description of change in adjusted TRPA level over time. A linear polynomial specification was determined to be of better fit. As such, linear latent class modelling was performed, starting with a standard one-class heterogeneous mixed model where all subjects are assumed to follow the same underlying trajectory over time, and increasing the number of latent classes progressively. Linear models with fixed slopes and intercepts, random intercepts and fixed slopes, and random intercepts and slopes were considered.

For each latent class model, posterior class-membership probabilities were used to determine the posterior classification of participants, as well as goodness-of-fit. The choice of best model was also based upon BIC values,(2). Literature advises that for an improvement to be considered significant a 10 point reduction of BIC was required in the competing model.(3) The proportion of subjects classified in each class with a posterior probability above a threshold of 0.8 was retrieved, this value indicative of the proportion of subjects unambiguously classified within each latent class. Mean posterior probabilities of belonging to the latent class were also retrieved, values between 0.8 and 1 were considered good. The best fitting model was determined to be the two-class linear model with random intercepts and slopes. Linear mixture model fit is shown in Table S2.

| Table S2. Latent class model fit | | | | | | |
| --- | --- | --- | --- | --- | --- | --- |
| Linear model | Number of latent classes | Log-likelihood | BIC | % Participants per class | Mean posterior probabilities | Posterior probabilities  > 0.8 (%) |
| Fixed intercepts & slopes | 1 | -7480.7 | 14981.0 | 100 | n.a. | n.a. |
|  | 2 | -7374.3 | 14787.9 | 6.1 / 93.9 | 0.90 / 0.98 | 76.7 / 97.4 |
|  | 3 | -7374.3 | 14807.6 | 93.6 / 6.4 / 0.0 | 0.72 / 0.88 /n.a. | 0.0 /73.3 / n.a. |
| Random intercepts,  fixed slopes | 1 | -7475.8 | 14977.7 | 100 | n.a. | n.a. |
|  | 2 | -7359.9 | 14772.2 | 92.0 / 8.0 | 0.97 / 0.86 | 96.4 / 64.3 |
|  | 3 | -7230.6 | 14539.8 | 4.7 / 84.2 / 11.1 | 0.91 / 0.95 / 0.87 | 81.8 / 95.3 / 74.4 |
| Random intercepts & slopes | 1 | -7471.7 | 14982.6 | 100 | n.a. | n.a. |
|  | **2** | **-7158.6** | **14382.8** | **26.5 / 73.5** | **0.94 / 0.92** | **87.63 / 89.92** |
|  | 3 | -7102.5 | 14296.8 | 67.4 / 6.3 / 26.3 | 0.88 / 0.86 / 0.85 | 82.9 / 65.9 / 75.7 |
| n.a – not applicable | | | | | | |

**Supplementary Analysis**

*Continuous childhood and adulthood adjusted transport-related physical activity*

Additional analysis was performed to assess the relationship between continuous childhood adjusted TRPA level (min/week) and adult TRPA latent class membership (i.e., class 1, class 2). Logistic regression was performed amongst 658 participants with observations of both adjusted childhood TRPA and adult TRPA class membership. This analysis found no significant relationship (odds ratio = 1.00; 95% confidence interval = 0.99 – 1.01) between childhood adjusted TRPA (min/week) and adulthood TRPA class membership.

| Table S3. Comparison of childhood characteristics of included and excluded participants | | |
| --- | --- | --- |
|  | Included  N=658 | Excluded  N=7480 |
| Age, mean (SD) | 11.9 (2.0) | 10.8 (2.6) |
| Sex (male), % (n) | 47.6 (313) | 50.9 (3994) |
| Body mass index (kg/m^2^),  mean (SD) | 18.5 (2.6) | 18.2 (2.9) |
| Self-reported health, % (n) | N=658 | N=5749 |
| Excellent | 37.2 (245) | 35.3 (2031) |
| Very good | 42.6 (280) | 43.7 (2510) |
| Good | 19.3 (127) | 19.6 (1127) |
| Fair & Poor | 0.9 (6) | 1.4 (81) |
| Teacher-reported scholastic ability, % (n) | N=658 | N=7303 |
| Excellent | 13.1 (86) | 9.0 (657) |
| Very good | 36.5 (240) | 27.0 (1971) |
| Good | 38.3 (252) | 41.4 (3027) |
| Fair | 10.5 (69) | 17.4 (1267) |
| Poor | 1.7 (11) | 5.2 (381) |
| Smoking duration, % (n) | N=658 | N=5712 |
| “I don’t smoke” | 90.1 (593) | 85.8 (4899) |
| “Just started” | 2.1 (14) | 2.5 (145) |
| 1-6 months | 1.8 (12) | 3.2 (182) |
| 7-12 months | 1.4 (9) | 2.2 (124) |
| 1-2 years | 2.4 (16) | 2.9 (165) |
| >2 years | 2.2 (14) | 3.4 (197) |
| ASHFS = Australian Schools Health and Fitness Survey,  Included = Included in the analyses of this study,  Excluded = Excluded from the analyses of this study,  SD = Standard Deviation | | |

**References**

1. Social Science Data Archives. Census of Population and Housing, 1986: Socio Economic Status Indicator File. User's Guide for the Machine-Readable Data File (SSDA Census Series). Canberra: Social Science Data Archives. ; 1998.

2. Proust-Lima C, Philipps V, Liquet B. Estimation of Extended Mixed Models Using Latent Classes and Latent Processes: the R package lcmm. 2015.

3. Nylund KL, Asparouhov T, Muthén B. Deciding on the number of classes in latent class analysis and growth mixture modeling: A Monte Carlo simulation study. Struc Equa Modeling Multdiscip J. 2007;14(4):535–69.
